# Balancing the functions of DNA extracellular traps in intracellular parasite infections: implications for host defense, disease pathology and therapy

**DOI:** 10.1038/s41419-023-05994-8

**Published:** 2023-07-20

**Authors:** Carolina Cattoni Koh, Kenneth J. Gollob, Walderez O. Dutra

**Affiliations:** 1 Morphology Dept, Universidade Federal de Minas Gerais, Belo Horizonte, MG Brazil; 2 National Institute for Science & Technology in Tropical Diseases – INCT-DT, Belo Horizonte, MG Brazil; 3 Albert Einstein Israelite Hospital, São Paulo, SP Brazil

**Keywords:** Cell death and immune response, Chronic inflammation

## Abstract

The release of DNA to the extracellular milieu is a biological process referred to as etosis, which is involved in both physiological and pathological functions. Although the release of DNA extracellular traps (ETs) was initially attributed to innate immune cells such as neutrophils, eosinophils, and macrophages, recent studies have shown that T cells, as well as non-immune cells, are capable of releasing ETs. These structures were described primarily for their potential to trap and kill pathogens, presenting an important strategy of host defense. Intriguingly, these functions have been associated with intracellular pathogens such as the parasites *Leishmania* sp. and *Trypanosoma cruzi*, causative agents of leishmaniasis and Chagas disease, respectively. These are two devastating tropical diseases that lead to thousands of deaths every year. In an apparent contradiction, ETs can also induce and amplify inflammation, which may lead to worsening disease pathology. This has prompted the concept of targeting ETs’ release as a means of controlling tissue destruction to treat human diseases. What is the best approach to prevent disease severity: inducing ETs to kill pathogens or preventing their release? In this Perspective article, we will discuss the importance of understanding ETs released by different cell types and the need to balance their potentially complementary functions. In addition, we will explore other functions of ETs and their translational applications to benefit individuals infected with intracellular parasites and other pathogens. Ultimately, a better understanding of the role of ETs in disease pathogenesis will provide valuable insights into developing novel therapies for human diseases.

## Introduction

Extracellular traps (ETs) are considered a form of cell death [1] and are mainly composed of DNA, proteins, and other cytoplasmic components released by cells. These structures have a diameter of 15–17 nm and globular domains of ~25 nm. Transmission electron microscopy analysis of cross-sections of these traps revealed that they are not enclosed by membranes [2–4]. ETs are released by cells through a process known as "Etosis", which involves the activation of a series of intracellular signaling pathways leading to DNA de-condensation and its release into the extracellular environment [1, 5–7]. Considerable research has been conducted regarding ETs since their discovery, but we still do not fully understand the process of their formation and how to control their release in vivo.

Etosis was initially described in human neutrophils [2] and has been extensively studied in these cells. Other cells of the innate response, such as eosinophils [8, 9], mast cells [10–15], monocytes and macrophages [14, 16, 17], basophils [18], and microglia [19] can also release ETs. Interestingly, whereas in most cells ETs are composed of nuclear DNA, eosinophils and basophils can release ETs composed of mitochondrial DNA [20, 21]. Importantly, it was recently demonstrated that CD8 + T cells [22], Th17 clones of CD4+ T cells [23], as well as B cells [24], all related to adaptive responses, can also release ETs. In addition to human cells, it has been shown that cells from many other living species such as mice [25–27], cats [28], dogs [29–31], sheep [32], bovines [33], horses [34], fish [20], chickens [35], insects [36], and plants [37] are also capable of releasing ETs. This wide variety of species in which ETs have been found shows that etosis is a mechanism conserved across species.

DNA is the main component responsible for the ability of ETs to capture and trap microorganisms [2, 38, 39]. The proteins present in ETs include histones and enzymes [2, 17, 40] such as elastase, which can degrade the cell walls of captured pathogens, implicating ETs in their elimination [41]. Thus, trapping and killing pathogens, the very first function attributed to ETs, is a coordinated effort of their many components. The role of ETs in combating extracellular pathogens such as bacteria [2, 15] and fungi [9, 42] is clearly an important defense mechanism. But the release of these structures can also be triggered by intracellular pathogens such as viruses [43, 44] and protozoan parasites [21, 45–49]. ETs can indeed trap and kill them while in their likely brief extracellular exposure. Despite significant advances in recent years in elucidating the release, composition, and functions of extracellular traps (ETs), the precise mechanisms underlying this process and the molecules that initiate their release remain incompletely understood. This knowledge gap is partly attributed to the diverse array of organisms that can activate ETs. These gaps represent critical areas of interest, as they offer potential avenues for developing novel strategies for controlling pathogenic infections and disease pathologies.

Comparing ET formation among infection models is challenging due to pathogen-specific and host-specific factors, limited data availability, and the lack of standardized methodologies. Standardization and collaborative research are crucial for advancing our understanding of ET formation in diverse infections. However, most studies on ETs have been conducted in neutrophils. These cells, in addition to being capable of forming extracellular DNA traps, are also capable of phagocytosing microorganisms. Therefore, the decision of neutrophils to generate NETs instead of phagocytosis is a crucial but still unknown point. This decision appears to be the result of a combination of multiple signals, including adhesive, metabolic, and activation conditions of the cells, environmental stimuli, and, importantly, the size and signals derived from the stimulating particle [50]. Some authors suggest that the size of the stimulating particle is important for the polarization of these two mechanisms [51]. It has been suggested that large particles, such as parasites, would induce NET formation, while small particles, such as bacteria and viruses, should be eliminated by phagocytosis. However, it has been demonstrated that both bacteria and viruses are capable of inducing cells to release ETs [2], while parasites, in addition to inducing NETs, can be phagocytized, as seen in studies with *Leishmania* sp and *T. cruzi* [21, 45–49]. The study by Sousa-Rocha D et al. in 2015 demonstrated that soluble *Trypanosoma cruzi* antigens as well as dead parasites are capable of inducing neutrophils to undergo Etosis [21]. Thus, although these parasites are obligatory intracellular pathogens, the interaction and activation necessary for NETosis occur mostly outside the cell. Table 1 summarizes the organisms in which the occurrence of ETs have been described, as well as the cellular source of ETs, and the stimulus that induced it formation.

**Table 1:** Summary of organism, cell origin and stimulus of extracellular DNA trap release.

Organism	ETs released by	Stimulated by	Classification	Type of infection in vivo	References
Bovine	Neutrophil	*Toxoplasma gondii*	Protozoa	Intracellular Obligatory	[31]
Cats	Neutrophil	*Gammaretrovirus*	Virus	Intracellular Obligatory	[26]
Chicken	Heterophil	Chemical Stimuli	–	Extracellular	[34]
Dogs	Granulocyte	*Trypanosoma cruzi*	Protozoa	Intracellular Obligatory	[29]
Opossum	Granulocyte	*Trypanosoma cruzi*	Protozoa	Intracellular Obligatory	[29]
Dogs	Neutrophil	*Toxoplasma gondii*	Protozoa	Intracellular Obligatory	[28]
Fish	Erythrocyte	Chemical Stimuli	–	–	[33]
Horse	Neutrophil	Chemical Stimuli	–	–	[32]
Human	Neutrophil	*Candida albicans*	Fungi	Extracellular	[38, 41]
Human	CD4+ cells	*Cutibacterium acnes*	Bacteria	Intracellular Facultative	[21]
Human	Eosinophil	*Escherichia coli*	Bacteria	Intracellular Facultative	[8]
Human	Eosinophil	*Aspergillus fumigatus*	Fungi	Intracellular Facultative	[9]
Human	Mast cell	*Listeria monocytogenes*	Bacteria	Intracellular Facultative	[10]
Human	Microglia	*Escherichia coli*	Bacteria	Intracellular Facultative	[19]
Human	Monocyte/ Macrophage	Chemical Stimuli	–	–	16[]
Human	Neutrophil	*Staphylococcus aureus*	Bacteria	Intracellular Facultative	[1, 2]
Human	Neutrophil	*Staphylococcus aureus*	Bacteria	Intracellular Facultative	[84]
Human	Neutrophil	*Leishmania amazonensis*	Protozoa	Intracellular Obligatory	[45, 59]
Human	Neutrophil	*Leishmania infantum*	Protozoa	Intracellular Obligatory	[44]
Human	Neutrophil	*Leishmania donovani*	Protozoa	Intracellular Obligatory	[63]
Human	Neutrophil	*Leishmania major*	Protozoa	Intracellular Obligatory	[63]
Human	Neutrophil	*SARS-CoV-2*	Virus	Intracellular Obligatory	[42, 85]
Human	Neutrophil	Chemical Stimuli	–		[40]
Human	CD8+, CD4+ cells	Chemical Stimuli	–		[20]
Insect	Hemocyte	*Pseudomonas entomophila*	Bacteria	Extracellular	[35]
Mouse	Basophil	*Nippostrongylus brasiliensis*	Nematoda	Extracellular	[18]
Mouse	Mast cell	*Mycobacterium bovis*	Bacteria	Intracellular Facultative	[13]
Mouse	Microglia	*Escherichia coli*	Bacteria	Intracellular Facultative	[19]
Mouse	Neutrophil	*Influenza virus*	Virus	Intracellular Obligatory	[43]
Mouse	Neutrophil	*Candida albicans*	Fungi	Intracellular Facultative	[38, 41]
Plant	Root cells	Unstimulated	–	–	[36]
Sheep	Neutrophil	*Streptococcus uberis*	Bacteria	Extracellular	[30]

## ETs in intracellular parasite infections

Most studies regarding the relationship of ETs and protozoan parasites were performed using *Leishmania*, the causative agent of leishmaniasis, a spectrum of diseases ranging from tegumentary to deadly visceral forms [52, 53]. *Leishmania* is transmitted to humans through the bite of an infected hematophagous female phlebotomine sandfly during her blood meal [54]. Amongst the tegumentary forms, cutaneous leishmaniasis is the most common manifestation and is characterized by single (localized, CL) or multiple (disseminated, DL) skin sores [55], while mucosal leishmaniasis (ML) mainly affects nasopharyngeal tissues [55]. These forms are mainly associated with *L. braziliensis* and *L. amazonensis* species in endemic areas of the Americas, where it is highly prevalent [56]. Visceral leishmaniasis (VL), caused mainly by *L. donovani* and *L. chagasi*, is the most severe form of the disease and can be fatal if not diagnosed early, and properly treated. It is estimated that there are 30,000 new cases of VL and over 1 million new cases of CL each year [57], and that more than 1 billion people are at risk of infection [58]. These diseases disproportionately affect economically and socially vulnerable populations, causing significant societal and economic impacts. Therefore, concerted efforts toward their control are of utmost importance.

Regardless of the species of *Leishmania*, two main stages of the parasite have been defined: amastigotes and promastigotes. Amastigotes typically reside inside the macrophages of the vertebrate host, while promastigotes are found mainly in the phlebotomine vector [57], and are the form transmitted during the sandfly’s bloodmeal.

The first report of the interaction between extracellular traps (ETs) and *Leishmania sp*. demonstrated that *L. amazonensis* promastigotes were ensnared in DNA, elastase, and histone-containing neutrophil extracellular traps (NETs), which exhibited leishmanicidal properties [46]. Moreover, immunofluorescence analysis of biopsies from patients with CL infected with *L. amazonensis* indicated the presence of DNA and elastase-containing structures, suggestive of NETs in vivo [46]. This finding was confirmed in a subsequent study by Morgado et al. [48]. Subsequent studies have revealed the crucial role of PI3Kinase isoforms in *L. amazonensis*-induced NETosis [59]. Specifically, it was demonstrated that PI3Kγ activates a reactive oxygen species (ROS)-dependent NETosis, whereas PI3Kδ induces a ROS-independent pathway regulated by intracellular calcium. These findings point to the potential of targeting the PI3K pathway as a strategy to control NET formation triggered by *L. amazonensis*.

It is interesting to note that while *L. amazonensis* is vulnerable to NETs, *L. infantum* is resistant to them. Although *L. infantum* is capable of inducing NET release, it can evade NET-mediated killing via 3′-nucleotidase/nuclease activity, revealing a new function for this enzyme [45]. It is unclear whether the susceptibility or resistance of *L. amazonensis* and *L. infantum*, respectively, to NET-mediated killing is directly linked to disease severity. Nevertheless, it is worth noting that the susceptible *L. amazonensis* is associated with milder forms of leishmaniasis, whereas the resistant *L. infantum* causes the severe and potentially fatal VL. Interestingly, molecules related to NETs are differentially regulated at different stages of *L. infantum* infection, with significant differences observed between patients with visceral leishmaniasis and asymptomatic individuals. These observations suggest that NETs may have distinct roles depending on the clinical stage of infection and may provide useful biomarkers for better characterizing asymptomatic infections in endemic regions [60].

The observation of ETs in lesions of CL and ML patients caused by *L. braziliensis* was a surprising finding, given the low number of polymorphonuclear cells and the predominance of mononuclear infiltrates in these lesions [61]. Koh et al. demonstrated the presence of CD8-derived ETs in lesions from patients with CL and ML. These ETs were found to co-localize with CD107+ vesicles and were correlated with disease progression and severity. In vitro studies showed that CD8-derived ETs contained CD107+ vesicles and, in a live video, were observed to mediate the death of neighboring cells. This study proposed a novel function for CD8-derived ETs, namely, the delivery of cytotoxic granules to target cells, suggesting a new mechanism of cytotoxicity that operates independently of cell-to-cell contact [25].

Recent studies demonstrated that the saliva of the *Leishmania sp*. vector, *Lutzomyia longipalpis*, contains a potent nuclease that digests NETs, thereby enabling parasites to escape NET-mediated killing [62]. Conversely, another study by Gabriel and colleagues showed that NETs may contribute to the retention of *L. donovani* promastigotes at the site of inoculation, facilitating their uptake by mononuclear phagocytes [63].

*Trypanosoma cruzi*, a protozoan that causes Chagas disease (CD), which affects millions of people worldwide, mainly in Latin America [57], is another intracellular parasite that can trigger the release of ETs. *T. cruzi* belongs to the kinetoplastid family, the same family as *Leishmania*. *T. cruzi* causes a lifelong infection, and at least 30% of infected individuals develop one of the most severe heart diseases reported, which leads to thousands of deaths and disabilities annually [64]. While blood transfusion, organ transplantation, infected food, and mother-to-child transmission are important forms of transmission, *T. cruzi* is mainly transmitted by contact with the contaminated excreta of a triatomine vector [65]. Trypomastigotes, the infective form, are internalized by several host cells, including monocytes and muscle cells, and transform into amastigote forms. These forms replicate and differentiate back into trypomastigotes, rupturing the cells and being released to be internalized by other cells [66].

*T. cruzi*, like *Leishmania*, can induce the release of NETs, which are composed of DNA, histones, and elastase [46]. This release of NETs was shown to be dose and time-dependent and also required the generation of reactive oxygen species. It was found that antibodies against Toll-like receptors 2 and 4 decreased the release of NETs, and both live and dead parasites were able to induce their release. Interestingly, the induction of NETs increased the number of amastigotes, suggesting that it may influence increasing parasite replication or decreasing the release of trypomastigote forms. These findings provide new insights into the interaction between parasites and NETs and suggest that contact with NETs during Chagas disease may limit infection by affecting the parasite’s infectivity and pathogenicity [21].

*T. cruzi* also induces ET formation by dog and opossum neutrophils. While the NETs were decorated with the protease elastase, it was suggested that the parasite efficiently evades ET-mediated killing since *T. cruzi* can survive in these hosts for years [31]. The saliva of blood-feeding arthropods, which include the triatomine vector of *T. cruzi*, contains proteins that exhibit high-affinity binding to prostanoids such as TXA2. In vitro studies have shown that these proteins can prevent platelet-mediated NET formation and may contribute to antithrombotic effects in vivo [67].

The pathology associated with Chagas disease and several forms of leishmaniasis is predominantly inflammatory. Koh et al. found a significant correlation between CD8-derived ETs and the progression and severity of tegumentary leishmaniasis. The frequency of CD8-derived ETs was higher in ulcerated CL lesions compared to early non-ulcerated ones, and in ML lesions compared to CL lesions. The ML form is characterized by an intense, uncontrolled inflammatory response, with high expression of TNF and IFN-gamma, and low expression of IL-10 receptor by inflammatory cells [68]. It is possible that CD8-derived ETs induced and exacerbated the inflammatory reaction and tissue destruction, but further research is needed to confirm this hypothesis. Analysis of the inflammatory infiltrate present in the myocardium of Chagas disease cardiomyopathy patients has shown an abundance of CD8+ cells expressing cytotoxic molecules and inflammatory cytokines [69–71]. However, it remains unclear if these CD8 cells or any other cell type in the infiltrate can release ETs. Importantly, previous research has shown a link between ETs and cardiovascular diseases such as atrial fibrillation [72], acute myocardial infarction [73], and hypertrophic remodeling of the myocardium [74], indicating that this mechanism could also be involved in Chagas disease.

## Targeting ETs to treat human diseases

The formation and release of extracellular traps (ETs) are complex cellular processes that involve the activation of various intracellular signaling pathways often associated with the inflammatory response. For instance, the activation of phosphoinositide 3-kinase (PI3K) and the generation of reactive oxygen species (ROS) have been implicated in this process [75]. The DNA present in ETs can stimulate specific receptors present in immune cells, including Toll-like receptor 9 (TLR9), which can trigger a signaling cascade leading to the production of inflammatory cytokines, such as IL-1β, IL-6, and TNF-α [76, 77]. Histones, which are also present in ETs, can engage membrane receptors and activate immune cells, thus contributing to the inflammatory response and inducing the production of inflammatory cytokines [78]. Therefore, ETs are involved in the inflammatory immune response, and their excessive release can lead to chronic inflammation and tissue damage. For example, in cases of sepsis, a severe infection that can lead to multiple organ failure, excessive ETs release can contribute to the destruction of surrounding tissues [79]. Similarly, in parasitic diseases such as those discussed above, the release of ETs can lead to chronic inflammation and tissue damage. Hence, a thorough understanding of the mechanisms underlying ET formation and release is essential to identify potential therapeutic targets for the treatment of inflammatory diseases.

Several therapeutic approaches have been considered to modulate the effects of ETs. Table 2 summarizes some of the strategies that have been employed to inhibit the production or release of ETs, showing their mechanism of action and potential applications. However, it is important to note that while controlling the activation of ET-releasing cells through inhibition of inflammatory signals is a valid approach, these control strategies should ideally act locally to better target the ETs themselves and prevent their activities. Moreover, some studies have questioned whether the generation of ETs is a physiological event necessary for biological functions since they may also occur spontaneously in the absence of specific stimuli [22]. Therefore, it is crucial to evaluate the impact of ET inhibition on both physiological and pathological processes to avoid unintended consequences. Another important consideration is that the majority of inhibitors were evaluated to impede the formation of extracellular traps (ETs) specifically by neutrophils. Given that ETs can be released by various cell types, it is crucial to ascertain whether these inhibitors would exhibit an inhibitory effect on the release of ETs by other cell types.

**Table 2:** Potential targets to control the formation or release of extracellular DNA.

Mechanism of action	Target	Compound name	Effect on ET formation/release	Effect on inflammation	References
Inhibits PAD4 enzyme activity	PAD4	Cl-amidine, GSK484	Decreases	Decreases	[86, 87]
Degrades extracellular DNA	Extracellular DNA	DNase I	Decreases	Decreases	[84]
Inhibition of PI3K signaling pathway	PI3K	Wortmannin	Decreases	Decreases	[88–90]
Inhibits histone-mediated activation of neutrophils	Histones	Heparin	Decreases	Decreases	[91]
Inhibits ROS production	NADPH oxidase	Fucoidan, Apocynin, Baicalein	Decreases	Decreases	[85, 92–94]
Inhibits the phosphorylation of NF-κB p65 subunit	NF-κB p65	Anti-inflammatory drugs ASA, BAY-11-7082, and Ro 106-9920	Decreases	Decreases	[95]
Inhibits NET formation pores	Gasdermin D	Disulfiram	Decreases	Decreases	[96, 97]
Cytokine blockade	IL-1β, TNF-a, IL-6	Anakinra, Infliximab, Tocilizumab	Decreases	Decreases	[98–100]
Protease inhibition	NE	Prolastin, Sivelestat	Decreases	Decreases	[101, 102]

*PAD4* peptidylarginine deiminase 4, *ROS* reactive oxygen species, *NADPH oxidase* nicotinamide adenine dinucleotide phosphate oxidase, *NE* neutrophils elastase.

## Concluding remarks

**Fig. 1:**
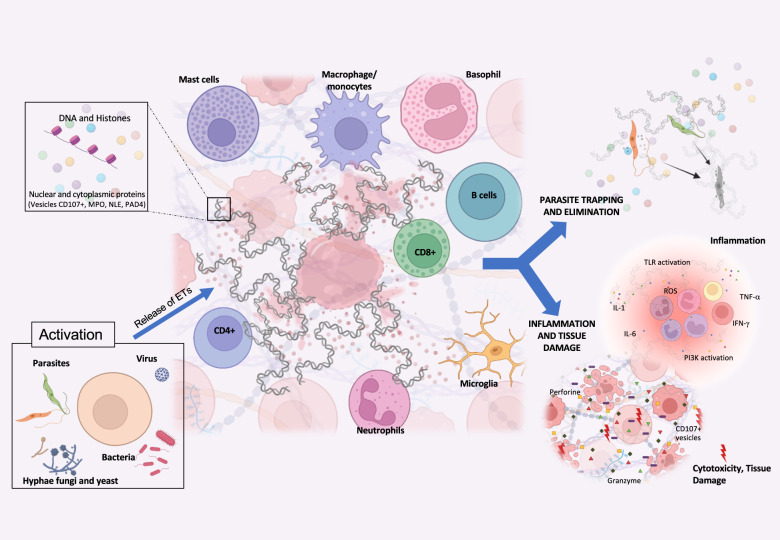
Illustration of the etosis process, in which cells release extracellular traps to capture and eliminate microorganisms. This process can be performed by several types of cells, including neutrophils and macrophages. Microorganisms that can activate this process include bacteria, viruses, fungi and protozoa. Extracellular traps are composed of DNA, histones, and various proteins depending on the cell type and stimulus. One of their functions is to capture and eliminate microorganisms such as *Leishmania sp*. and *T. cruzi*. However, extracellular traps can also cause inflammation and tissue damage by stimulating the local production of cytokines and other proinflammatory molecules. Understanding these processes may help identify targets for therapeutic intervention, offering new alternatives to treat human diseases.

The parasites *T. cruzi* and *Leishmania sp*. have undergone co-evolution with mammalian hosts for millions of years, acquiring sophisticated mechanisms to evade the host’s immune responses and persist in host tissues for prolonged periods. As a result, these parasites can cause chronic and debilitating diseases that significantly impact human health. Unfortunately, no vaccines for these diseases exist, and the available therapies are often limited by parasite resistance and serious side effects [80–83]. ETs possess both the ability to eliminate pathogens and to induce inflammation and tissue destruction, as demonstrated in Fig. 1, through complex cell activation mechanisms and functions. This concurrent occurrence of apparently opposing functions—parasite control and tissue destruction—prompts the question of whether to induce or inhibit the release of ETs to control infections and their consequences. Early ET release may benefit the host by clearing the pathogen, but interventions to control inflammation and pathology must be introduced subsequently. It is essential to conduct further research to determine the best timing for intervention and address critical questions such as: how parasites use ETs to evade host defenses, which specific molecules induce ET release in different diseases, whether this process depends on ligand-receptor interactions, and what are the consequences of inhibiting ET formation and release, given their potential physiological functions. Intracellular parasites are an excellent model for exploring these simultaneous and essential functions in these infections. By investigating the dual functions of ETs in host defense and pathology, new insights may emerge, leading to innovative strategies to combat these diseases.

## Data Availability

This article did not involve the generation or analysis of any datasets; therefore, data sharing is not relevant in this case.

## References

[1] Fuchs TA, Abed U, Goosmann C, Hurwitz R, Schulze I, Wahn V (2007). Novel cell death program leads to neutrophil extracellular traps. J Cell Biol.

[2] Brinkmann V, Reichard U, Goosmann C, Fauler B, Uhlemann Y, Weiss DS (2004). Neutrophil extracellular traps kill bacteria. Science..

[3] Hakkim A, Fuchs TA, Martinez NE, Hess S, Prinz H, Zychlinsky A (2011). Activation of the Raf-MEK-ERK pathway is required for neutrophil extracellular trap formation. Nat Chem Biol.

[4] Rohrbach AS, Slade DJ, Thompson PR, Mowen KA (2012). Activation of PAD4 in NET formation. Front Immunol.

[5] Brinkmann V, Zychlinsky A (2012). Neutrophil extracellular traps: is immunity the second function of chromatin?. J Cell Biol.

[6] Farhan A, Hassan G, Ali SHL, Yousaf Z, Shafique K, Faisal A (2023). Spontaneous NETosis in diabetes: a role of hyperglycemia mediated ROS and autophagy. Front Med (Lausanne).

[7] Tatsiy O, McDonald PP (2018). Physiological stimuli induce PAD4-dependent, ROS-independent NETosis, with early and late events controlled by discrete signaling pathways. Front Immunol.

[8] Yousefi S, Gold JA, Andina N, Lee JJ, Kelly AM, Kozlowski E (2008). Catapult-like release of mitochondrial DNA by eosinophils contributes to antibacterial defense. Nat Med.

[9] Muniz VS, Silva JC, Braga YAV, Melo RCN, Ueki S, Takeda M (2018). Eosinophils release extracellular DNA traps in response to *Aspergillus fumigatus*. J Allergy Clin Immunol.

[10] Campillo-Navarro M, Leyva-Paredes K, Donis-Maturano L, González-Jiménez M, Paredes-Vivas Y, Cerbulo-Vázquez A (2017). *Listeria monocytogenes* induces mast cell extracellular traps. Immunobiology..

[11] Garcia-Rodriguez KM, Bahri R, Sattentau C, Roberts IS, Goenka A, Bulfone-Paus S. Human mast cells exhibit an individualized pattern of antimicrobial responses. Immun Inflamm Dis. 2020:198–210. 10.1002/iid3.295. 10.1002/iid3.295 PMC7212193 32222064

[12] Jiménez M, Cervantes-García D, Córdova-Dávalos LE, Pérez-Rodríguez MJ, Gonzalez-Espinosa C, Salinas E (2021). Responses of mast cells to pathogens: beneficial and detrimental roles. Front Immunol.

[13] Naqvi N, Srivastava R, Naskar P, Puri N (2021). Mast cells modulate early responses to *Mycobacterium bovis* Bacillus Calmette-Guerin by phagocytosis and formation of extracellular traps. Cell Immunol.

[14] Pertiwi KR, de Boer OJ, Mackaaij C, Pabittei DR, de Winter RJ, Li X (2019). Extracellular traps derived from macrophages, mast cells, eosinophils and neutrophils are generated in a time-dependent manner during atherothrombosis. J Pathol.

[15] von Köckritz-Blickwede M, Nizet V (2009). Innate immunity turned inside-out: antimicrobial defense by phagocyte extracellular traps. J Mol Med (Berl).

[16] Chow OA, von Köckritz-Blickwede M, Bright AT, Hensler ME, Zinkernagel AS, Cogen AL (2010). Statins enhance formation of phagocyte extracellular traps. Cell Host Microbe.

[17] Jensen M, Thorsen NW, Hallberg LAE, Hägglund P, Hawkins CL (2023). New insight into the composition of extracellular traps released by macrophages exposed to different types of inducers. Free Radic Biol Med.

[18] Morshed M, Hlushchuk R, Simon D, Walls AF, Obata-Ninomiya K, Karasuyama H (2014). NADPH oxidase-independent formation of extracellular DNA traps by basophils. J Immunol.

[19] Agrawal I, Sharma N, Saxena S, Arvind S, Chakraborty D, Chakraborty DB (2021). Protocol for induction and characterization of microglia extracellular traps in murine and human microglia cells. STAR Protoc.

[20] Rinaldi G, Álvarez de Haro N, Fernando AJ, Desbois AP, Robb CT, et al. Fish Erythrocyte Extracellular Traps (FEETs) are an evolutionary conserved cellular process triggered by different stimuli. Fish Shellfish Immunol. 2023:108638. 10.1016/j.fsi.2023.108638. 10.1016/j.fsi.2023.108638 36842638

[21] Sousa-Rocha D, Thomaz-Tobias M, Diniz LF, Souza PS, Pinge-Filho P, Toledo KA (2015). *Trypanosoma cruzi* and Its Soluble Antigens Induce NET Release by Stimulating Toll-Like Receptors. PLoS ONE.

[22] Koh CC, Wardini AB, Vieira M, Passos LSA, Martinelli PM, Neves EGA (2020). Human CD8+ T Cells release extracellular traps co-localized with cytotoxic vesicles that are associated with lesion progression and severity in human leishmaniasis. Front Immunol.

[23] Nguyen NPN, Oparaugo NC, Ouyang K, Agak GW (2022). A protocol to detect human CD4+ T cell extracellular traps using scanning electron microscopy. STAR Protoc.

[24] Rocha Arrieta YC, Rojas M, Vasquez G, Lopez J (2017). The lymphocytes stimulation induced DNA release, a phenomenon similar to NETosis. Scand J Immunol.

[25] Veras FP, Gomes GF, Silva BMS, Caetité DB, Almeida CJLR, Silva CMS (2023). Targeting neutrophils extracellular traps (NETs) reduces multiple organ injury in a COVID-19 mouse model. Respir Res.

[26] Fadini GP, Menegazzo L, Rigato M, Scattolini V, Poncina N, Bruttocao A (2016). NETosis delays diabetic wound healing in mice and humans. Diabetes..

[27] Martinod K, Witsch T, Erpenbeck L, Savchenko A, Hayashi H, Cherpokova D (2017). Peptidylarginine deiminase 4 promotes age-related organ fibrosis. J Exp Med.

[28] Wardini AB, Guimarães-Costa AB, Nascimento MT, Nadaes NR, Danelli MG, Mazur C (2010). Characterization of neutrophil extracellular traps in cats naturally infected with feline leukemia virus. J Gen Virol.

[29] McQuinn ER, Smith SA, Viall AK, Wang C, LeVine DN (2020). Neutrophil extracellular traps in stored canine red blood cell units. J Vet Intern Med.

[30] Wei Z, Wang Z, Liu X, Wang C, Han Z, Wu D (2020). *Toxoplasma gondii* triggers neutrophil extracellular traps release in dogs. Front Cell Infect Microbiol.

[31] de Buhr N, Bonilla MC, Jimenez-Soto M, von Köckritz-Blickwede M, Dolz G (2018). Extracellular trap formation in response to *Trypanosoma cruzi* infection in granulocytes isolated from dogs and common opossums, natural reservoir hosts. Front Microbiol.

[32] Pisanu S, Cubeddu T, Pagnozzi D, Rocca S, Cacciotto C, Alberti A (2015). Neutrophil extracellular traps in sheep mastitis. Vet Res.

[33] Velásquez ZD, Peixoto R, Gärtner U, Hermosilla C, Taubert A, Conejeros I (2023). Dynamics of cell cycle proteins involved in *Toxoplasma gondii*-induced bovine NET formation. Front Immunol.

[34] Salinas C, Barriga K, Albornoz A, Alarcon P, Quiroga J, Uberti B (2023). Tamoxifen triggers the in vitro release of neutrophil extracellular traps in healthy horses. Front Vet Sci.

[35] Chuammitri P, Ostojić J, Andreasen CB, Redmond SB, Lamont SJ, Palić D (2009). Chicken heterophil extracellular traps (HETs): novel defense mechanism of chicken heterophils. Vet Immunol Immunopathol.

[36] Carrau T, Thümecke S, Silva LMR, Perez-Bravo D, Gärtner U, Taubert A (2021). The cellular innate immune response of the invasive pest insect Drosophila suzukii against *Pseudomonas entomophila* involves the release of extracellular traps. Cells.

[37] Chambard M, Plasson C, Derambure C, Coutant S, Tournier I, Lefranc B (2021). New insights into plant extracellular DNA. A study in soybean root extracellular trap. Cells.

[38] Bartneck M, Keul HA, Zwadlo-Klarwasser G, Groll J. Phagocytosis independent extracellular nanoparticle clearance by human immune cells. Nano Lett. 2010:59–63. 10.1021/nl902830x. 10.1021/nl902830x 19994869

[39] Urban CF, Ermert D, Schmid M, Abu-Abed U, Goosmann C, Nacken W, et al. Neutrophil extracellular traps contain calprotectin, a cytosolic protein complex involved in host defense against Candida albicans. PLoS Pathog. 2009:e1000639. 10.1371/journal.ppat.1000639. 10.1371/journal.ppat.1000639 PMC2763347 19876394

[40] Petretto A, Bruschi M, Pratesi F, Croia C, Candiano G, Ghiggeri G (2019). Neutrophil extracellular traps (NET) induced by different stimuli: a comparative proteomic analysis. PLoS ONE.

[41] Papayannopoulos V, Metzler KD, Hakkim A, Zychlinsky A (2010). Neutrophil elastase and myeloperoxidase regulate the formation of neutrophil extracellular traps. J Cell Biol.

[42] Urban CF, Reichard U, Brinkmann V, Zychlinsky A (2006). Neutrophil extracellular traps capture and kill Candida albicans yeast and hyphal forms. Cell Microbiol.

[43] Lebourgeois S, David A, Chenane HR, Granger V, Menidjel R, Fidouh N (2022). Differential activation of human neutrophils by SARS-CoV-2 variants of concern. Front Immunol.

[44] Hemmers S, Teijaro JR, Arandjelovic S, Mowen KA (2011). PAD4-mediated neutrophil extracellular trap formation is not required for immunity against influenza infection. PLoS ONE.

[45] Guimarães-Costa AB, DeSouza-Vieira TS, Paletta-Silva R, Freitas-Mesquita AL, Meyer-Fernandes JR, Saraiva EM (2014). 3′-nucleotidase/nuclease activity allows Leishmania parasites to escape killing by neutrophil extracellular traps. Infect Immun.

[46] Guimarães-Costa AB, Nascimento MT, Froment GS, Soares RP, Morgado FN, Conceição-Silva F (2009). *Leishmania amazonensis* promastigotes induce and are killed by neutrophil extracellular traps. Proc Natl Acad Sci USA.

[47] Hurrell BP, Regli IB, Tacchini-Cottier F (2016). Different Leishmania species drive distinct neutrophil functions. Trends Parasitol.

[48] Morgado FN, Nascimento MT, Saraiva EM, de Oliveira-Ribeiro C, Madeira Mde F, da Costa-Santos M (2015). Are neutrophil extracellular traps playing a role in the parasite control in active American tegumentary leishmaniasis lesions?. PLoS ONE.

[49] Muñoz-Caro T, Machado Ribeiro da Silva L, Rentería-Solis Z, Taubert A, Hermosilla C (2016). Neutrophil extracellular traps in the intestinal mucosa of Eimeria-infected animals. Asian Pac J Trop Biomed.

[50] Manfredi AA, Ramirez GA, Rovere-Querini P, Maugeri N (2018). The neutrophil’s choice: phagocytose vs make neutrophil extracellular traps. Front Immunol.

[51] Branzk N, Lubojemska A, Hardison SE, Wang Q, Gutierrez MG, Brown GD (2014). Neutrophils sense microbe size and selectively release neutrophil extracellular traps in response to large pathogens. Nat Immunol.

[52] Cáceres-Dittmar G, Sánchez MA, Oriol O, Kraal G, Tapia FJ (1992). Epidermal compromise in American cutaneous leishmaniasis. J Invest Dermatol.

[53] Evans T, Reis Mde F, de Alencar JE, Naidu TG, de Jesus JA, McAuliffe JF (1985). American visceral leishmaniasis (kala-azar). West J Med.

[54] Killick-Kendrick R, Molyneux DH (1981). Transmission of leishmaniasis by the bite of phlebotomine sandflies: possible mechanisms. Trans R Soc Trop Med Hyg.

[55] Herwaldt BL (1999). Leishmaniasis. Lancet.

[56] World Health Organizaton. Leishmaniasis: Cutaneous and mucosal leishmaniasis [Internet]. Washington, D.C.: Pan American Health Organization; c2021 [cited 2023 Apr 14]. Available from: https://www.paho.org/en/topics/leishmaniasis/cutaneous-and-mucosal-leishmaniasis.

[57] World Health Organization. WHO - World Health Organization. [Internet]. Geneva: World Health Organization; [cited 2023 Apr 12]. Available from: https://www.who.int/.

[58] Wamai RG, Kahn J, McGloin J, Ziaggi G. Visceral leishmaniasis: a global overview. J Glob Health Sci. 2020:e3. 10.35500/jghs.2020.2.e3.

[59] DeSouza-Vieira T, Guimarães-Costa A, Rochael NC, Lira MN, Nascimento MT, Lima-Gomez PS (2016). Neutrophil extracellular traps release induced by Leishmania: role of PI3Kγ, ERK, PI3Kσ, PKC, and [Ca2+]. J Leukoc Biol.

[60] Gardinassi LG, DeSouza-Vieira TS, da Silva NO, Garcia GR, Borges VM, Campos RNS (2017). Molecular signatures of neutrophil extracellular traps in human visceral leishmaniasis. Parasit Vectors.

[61] Faria DR, Souza PE, Durães FV, Carvalho EM, Gollob KJ, Machado PR (2009). Recruitment of CD8(+) T cells expressing granzyme A is associated with lesion progression in human cutaneous leishmaniasis. Parasite Immunol.

[62] Chagas AC, Oliveira F, Debrabant A, Valenzuela JG, Ribeiro JM, Calvo E (2014). Lundep, a sand fly salivary endonuclease increases *Leishmania* parasite survival in neutrophils and inhibits XIIa contact activation in human plasma. PLoS Pathog.

[63] Gabriel C, McMaster WR, Girard D, Descoteaux A (2010). *Leishmania donovani* promastigotes evade the antimicrobial activity of neutrophil extracellular traps. J Immunol.

[64] Torres RM, Correia D, Nunes MDCP, Dutra WO, Talvani A, Sousa AS (2022). Prognosis of chronic Chagas heart disease and other pending clinical challenges. Mem Inst Oswaldo Cruz.

[65] Zeledón R, Rabinovich JE (1981). Chagas’ disease: an ecological appraisal with special emphasis on its insect vectors. Annu Rev Entomol.

[66] Tyler KM, Engman DM (2001). The life cycle of *Trypanosoma cruzi* revisited. Int J Parasitol.

[67] Mizurini DM, Aslan JS, Gomes T, Ma D, Francischetti IM, Monteiro RQ (2015). Salivary Thromboxane A2-Binding Proteins from Triatomine Vectors of Chagas Disease Inhibit Platelet-Mediated Neutrophil Extracellular Traps (NETs) Formation and Arterial Thrombosis. PLoS Negl Trop Dis.

[68] Faria DR, Gollob KJ, Barbosa J, Schriefer A, Machado PR, Lessa H (2005). Decreased in situ expression of interleukin-10 receptor is correlated with the exacerbated inflammatory and cytotoxic responses observed in mucosal leishmaniasis. Infect Immun.

[69] Argüello RJ, Vigliano C, Cabeza-Meckert P, Viotti R, Garelli F, Favaloro LE (2014). Presence of antigen-experienced T cells with low grade of differentiation and proliferative potential in chronic Chagas disease myocarditis. PLoS Negl Trop Dis.

[70] Reis DD, Jones EM, Tostes S, Lopes ER, Gazzinelli G, Colley DG (1993). Characterization of inflammatory infiltrates in chronic chagasic myocardial lesions: presence of tumor necrosis factor-alpha+ cells and dominance of granzyme A+, CD8+ lymphocytes. Am J Trop Med Hyg.

[71] Higuchi Mde L, Gutierrez PS, Aiello VD, Palomino S, Bocchi E, Kalil J (1993). Immunohistochemical characterization of infiltrating cells in human chronic chagasic myocarditis: comparison with myocardial rejection process. Virchows Arch a Pathol Anat Histopathol.

[72] Arroyo AB, de Los Reyes-García AM, Rivera-Caravaca JM, Valledor P, García-Barberá N, Roldán V (2018). MiR-146a regulates neutrophil extracellular trap formation that predicts adverse cardiovascular events in patients with atrial fibrillation. Arterioscler Thromb Vasc Biol.

[73] Novotny J, Oberdieck P, Titova A, Pelisek J, Chandraratne S, Nicol P (2020). Thrombus NET content is associated with clinical outcome in stroke and myocardial infarction. Neurology.

[74] Becker RC, Owens AP, Sadayappan S (2020). Tissue-level inflammation and ventricular remodeling in hypertrophic cardiomyopathy. J Thromb Thrombolysis.

[75] Sharma AK, Taneja G, Khanna D, Rajput SK (2015). Reactive oxygen species: friend or foe?. RSC Adv.

[76] Kawasaki T, Kawai T (2014). Toll-like receptor signaling pathways. Front Immunol.

[77] Keshari RS, Jyoti A, Dubey M, Kothari N, Kohli M, Bogra J (2012). Cytokines induced neutrophil extracellular traps formation: implication for the inflammatory disease condition. PLoS ONE.

[78] Saffarzadeh M, Juenemann C, Queisser MA, Lochnit G, Barreto G, Galuska SP (2012). Neutrophil extracellular traps directly induce epithelial and endothelial cell death: a predominant role of histones. PLoS ONE.

[79] Czaikoski PG, Mota JM, Nascimento DC, Sônego F, Castanheira FV, Melo PH (2016). Neutrophil extracellular traps induce organ damage during experimental and clinical sepsis. PLoS ONE.

[80] Bahia-Oliveira LM, Gomes JA, Cançado JR, Ferrari TC, Lemos EM, Luz ZM (2000). Immunological and clinical evaluation of chagasic patients subjected to chemotherapy during the acute phase of *Trypanosoma cruzi* infection 14-30 years ago. J Infect Dis.

[81] Cançado JR (1999). Criteria of Chagas disease cure. Mem Inst Oswaldo Cruz.

[82] Kedzierski L, Zhu Y, Handman E (2006). Leishmania vaccines: progress and problems. Parasitology..

[83] Lee BY, Bacon KM, Connor DL, Willig AM, Bailey RR (2010). The potential economic value of a *Trypanosoma cruzi* (Chagas disease) vaccine in Latin America. PLoS Negl Trop Dis.

[84] Lewis HD, Liddle J, Coote JE, Atkinson SJ, Barker MD, Bax BD (2015). Inhibition of PAD4 activity is sufficient to disrupt mouse and human NET formation. Nat Chem Biol.

[85] Cross AL, Hawkes J, Wright HL, Moots RJ, Edwards SW (2020). APPA (apocynin and paeonol) modulates pathological aspects of human neutrophil function, without supressing antimicrobial ability, and inhibits TNFα expression and signalling. Inflammopharmacology.

[86] Pilsczek FH, Salina D, Poon KK, Fahey C, Yipp BG, Sibley CD (2010). A novel mechanism of rapid nuclear neutrophil extracellular trap formation in response to *Staphylococcus aureus*. J Immunol.

[87] Huang TH, Hsieh PW, Chen TJ, Tsai HJ, Cheng JC, Liao HR (2023). *Melastoma malabathricum* L. suppresses neutrophil extracellular trap formation induced by synthetic analog of viral double-stranded RNA associated with SARS-CoV-2 infection. Pathogens.

[88] Willis VC, Gizinski AM, Banda NK, Causey CP, Knuckley B, Cordova KN (2011). N-α-benzoyl-N5-(2-chloro-1-iminoethyl)-L-ornithine amide, a protein arginine deiminase inhibitor, reduces the severity of murine collagen-induced arthritis. J Immunol.

[89] Kimura H, Matsuyama Y, Araki S, Koizumi A, Kariya Y, Takasuga S (2018). The effect and possible clinical efficacy of in vivo inhibition of neutrophil extracellular traps by blockade of PI3K-gamma on the pathogenesis of microscopic polyangiitis. Mod Rheumatol.

[90] de Carvalho Oliveira V, Tatsiy O, McDonald PP (2023). Phosphoinositol 3-kinase-driven NET formation involves different isoforms and signaling partners depending on the stimulus. Front Immunol.

[91] Arcaro A, Wymann MP (1993). Wortmannin is a potent phosphatidylinositol 3-kinase inhibitor: the role of phosphatidylinositol 3,4,5-trisphosphate in neutrophil responses. Biochem J.

[92] Hogwood J, Pitchford S, Mulloy B, Page C, Gray E (2020). Heparin and non-anticoagulant heparin attenuate histone-induced inflammatory responses in whole blood. PLOS ONE.

[93] Zhang FL, He Y, Zheng Y, Zhang WJ, Wang Q, Jia YJ (2014). Therapeutic effects of fucoidan in 6-hydroxydopamine-lesioned rat model of Parkinson’s disease: Role of NADPH oxidase-1. CNS Neurosci Ther.

[94] Moraes JA, Frony AC, Barcellos-de-Souza P, Menezes da Cunha M, Brasil Barbosa Calcia T, Benjamim CF (2019). Downregulation of microparticle release and pro-inflammatory properties of activated human polymorphonuclear neutrophils by LMW Fucoidan. J Innate Immun.

[95] Lapponi MJ, Carestia A, Landoni VI, Rivadeneyra L, Etulain J, Negrotto S (2013). Regulation of neutrophil extracellular trap formation by anti-inflammatory drugs. J Pharm Exp Ther.

[96] Sollberger G, Choidas A, Burn GL, Habenberger P, Di Lucrezia R, Kordes S (2018). Gasdermin D plays a vital role in the generation of neutrophil extracellular traps. Sci Immunol.

[97] Adrover JM, Carrau L, Daßler-Plenker J, Bram Y, Chandar V, Houghton S (2022). Disulfiram inhibits neutrophil extracellular trap formation and protects rodents from acute lung injury and SARS-CoV-2 infection. JCI Insight.

[98] Hudock KM, Collins MS, Imbrogno M, Snowball J, Kramer EL, Brewington JJ (2020). Neutrophil extracellular traps activate IL-8 and IL-1 expression in human bronchial epithelia. Am J Physiol Lung Cell Mol Physiol.

[99] Liberale L, Holy EW, Akhmedov A, Bonetti NR, Nietlispach F, Matter CM (2019). Interleukin-1β mediates arterial thrombus formation via NET-associated tissue factor. J Clin Med.

[100] Pérez-Sánchez C, Ruiz-Limón P, Aguirre MA, Jiménez-Gómez Y (2017). Arias-de la Rosa I, et al. Diagnostic potential of NETosis-derived products for disease activity, atherosclerosis and therapeutic effectiveness in Rheumatoid Arthritis patients. J Autoimmun.

[101] Tagami T, Tosa R, Omura M, Fukushima H, Kaneko T, Endo T (2014). Effect of a selective neutrophil elastase inhibitor on mortality and ventilator-free days in patients with increased extravascular lung water: a post hoc analysis of the PiCCO Pulmonary Edema Study. J Intensive Care.

[102] Sorrells S, Camprubi S, Griffin R, Chen J, Ayguasanosa J (2015). SPARTA clinical trial design: exploring the efficacy and safety of two dose regimens of alpha1-proteinase inhibitor augmentation therapy in alpha1-antitrypsin deficiency. Respir Med.

